# Predicting fruit consumption: the role of habits, previous behavior and mediation effects

**DOI:** 10.1186/1471-2458-14-730

**Published:** 2014-07-18

**Authors:** Hein de Vries, Sander M Eggers, Lilian Lechner, Liesbeth van Osch, Maartje M van Stralen

**Affiliations:** 1 Department of Health Education and Health Promotion, Research School Caphri, Maastricht University, 6200MD Maastricht, The Netherlands; 2 Department of Psychology, Open University of the Netherlands, 6419AT Heerlen, The Netherlands; 3 EMGO Institute for Health and Care Research and Department of Public and Occupational Health, VU University Medical Center, 1081 HV Amsterdam, The Netherlands

**Keywords:** Fruit consumption, Habit, Previous behavior, SRHI

## Abstract

**Background:**

This study assessed the role of habits and previous behavior in predicting fruit consumption as well as their additional predictive contribution besides socio-demographic and motivational factors. In the literature, habits are proposed as a stable construct that needs to be controlled for in longitudinal analyses that predict behavior. The aim of this study is to provide empirical evidence for the inclusion of either previous behavior or habits.

**Methods:**

A random sample of 806 Dutch adults (>18 years) was invited by an online survey panel of a private research company to participate in an online study on fruit consumption. A longitudinal design (*N* = 574) was used with assessments at baseline and after one (T2) and two months (T3). Multivariate linear regression analysis was used to assess the differential value of habit and previous behavior in the prediction of fruit consumption.

**Results:**

Eighty percent of habit strength could be explained by habit strength one month earlier, and 64% of fruit consumption could be explained by fruit consumption one month earlier. Regression analyses revealed that the model with motivational constructs explained 41% of the behavioral variance at T2 and 38% at T3. The addition of previous behavior and habit increased the explained variance up to 66% at T2 and to 59% at T3. Inclusion of these factors resulted in non-significant contributions of the motivational constructs. Furthermore, our findings showed that the effect of habit strength on future behavior was to a large extent mediated by previous behavior.

**Conclusions:**

Both habit and previous behavior are important as predictors of future behavior, and as educational objectives for behavior change programs. Our results revealed less stability for the constructs over time than expected. Habit strength was to a large extent mediated by previous behavior and our results do not strongly suggest a need for the inclusion of both constructs. Future research needs to assess the conditions that determine direct influences of both previous behavior and habit, since these influences may differ per type of health behavior, per context stability in which the behavior is performed, and per time frame used for predicting future behavior.

## Background

Fruit consumption is associated with several health advantages, such as lowered heart disease risk and lower risks of cancer (e.g. cancers of the gastrointestinal and respiratory tracts) [1,2]. Fruits are recommended as an alternative, healthy snack for weight control and maintenance because of their low energy density. Dutch guidelines recommend eating at least two pieces of fruit per day, equal to 200 grams per day. However, these recommendations are not met by 90% of the Dutch adults, who have a much lower median consumption per day ranging from 61 to 145 grams [3].

Changing health behaviors, such as fruit consumption, often implies changing motivational beliefs and replacing old habits by new ones. Health education messages often address motivational beliefs, but they rarely explicitly address habits. A habit is a behavior that is frequently performed, has acquired a high degree of automaticity and is cued consistently in stable contexts [4]. Habits develop from actions that yield rewarding outcomes and/or are repeated. Habit formation is more likely to occur for activities that people perform in stable contexts (e.g., exercising, drinking milk) compared to less stable situations, such as mammography attendance [5,6]. Some scholars outline that future actions will be primarily guided by habits, rather than by beliefs, since habits do not require elaborate processes of reasoning [7]. For nutrition behaviors, the correlations with habit are around .45; habit alone can thus explain 20% of nutrition behavior [8]. Hence, habits are an important factor for understanding and changing fruit consumption [5,9-14].

Several studies emphasized the importance of previous behavior within the context of various health behaviors using longitudinal studies [15,16]. For this purpose, self-reported previous behavior and/or reported frequency of previous behavior are often used in research [5]. Yet, a difference between behavioral frequency and habit cannot be assessed without an independent measure of habit. Hence, Verplanken and Orbell [17] proposed the Self-Report Habit Index, a 12-item self-report instrument that measures different aspects of habitual behaviors, such as history of repetition, lack of awareness, difficulty to control, mental efficiency and a sense of self-description. Several studies have demonstrated its reliability and validity for various behaviors, and showed that habit contributes significantly to the prediction of future behavior over and above social cognition constructs such as attitudes, social influence beliefs and self-efficacy (see e.g. [17-20]). Yet, several studies that outlined the importance of habits and previous behavior were cross-sectional (see e.g. [10]) and did not always compare both constructs. Hence, several questions remain unanswered. This paper aims to discuss three of them.

First, given the nature of habits, one could expect more stability in measurements of habit over time than for assessments of behavior over time. Although habits are regarded as a behavioral tendency to repeat well-practiced acts in response to stable environmental cues [5], little research is available comparing the contributions of both factors and comparing the temporal stability of habit and behavior. Although environmental cues can prompt habitual behaviors, we can also foresee that the performance of a behavior is more directly subject to distortion due to personal, social and environmental stimuli (e.g. stress, a friend not eating fruit). These distortions may temporally influence execution of a behavior, but are not expected to influence a long established habit. Therefore, the first goal of this study is to assess the temporal stability of the two constructs using a longitudinal design.

The second point of interest concerns the role of previous behavior and habits within the context of motivational factors. The Theory of Planned Behavior [21] and the Reasoned Action Approach ([22] p. 22) assume that the influence of previous behavior (and habits) will be mediated via attitudes, social influence beliefs and self-efficacy [21,23]. Alternatively, an integrative model, developed in 1995 as a result of TPB and Social Cognition Theory [24], now referred to as the I-Change Model, proposed to include previous behavior in theoretical models, as it was noted that mediation of previous behavior via motivational factors did not occur as expected and revealed that the impact of habits and/or previous behavior was not always fully mediated by motivational factors [15,16,18,19,25-29]. Furthermore, evidence of the additional value of habit strength over previous behavior is limited and research is lacking in which both factors are assessed in a longitudinal study. The second goal is therefore to assess the different impacts of both previous behavior and habit strength of fruit consumption in conjunction with socio-demographic and motivational factors.

Along with the findings of Verplanken [30], we expect that habit and past behavior will independently predict future behavior. If, however, the role of previous behavior can be fully explained by habit, then the effect of previous behavior on fruit consumption should be mediated by habit strength. Our third goal is to assess this potential mediation effect.

## Methods

### Sample

A random sample of 806 Dutch adults (>18 years) was invited by an online survey panel of a private research company to participate in an online study on fruit consumption [31]. As this study did not involve medical ethical research no ethics approval was required as described in the Dutch Act for medical-scientific research in humans [32,33]. Participants were explained that confidentiality would be ensured, that the concerning study would comprise three measurements and that they would receive a small incentive (approximately € 3) after completing all three questionnaires. By activating a link in the invitation e-mail, participants were directed to the web page where they could fill out the questionnaire.

At the baseline measurement (T1), 574 respondents (71.0%) filled out the questionnaire on fruit consumption. At the first follow-up measurement one month later (T2), 498 respondents participated (87.1% of baseline), whereas a total of 434 respondents (75.9% of baseline) had completed all three questionnaires at the second follow-up measurement two months after baseline (T3).

### Measurements

Questionnaire items were based on the I-Change Model and questionnaires used in previous studies (see e.g. [31,34]) and assessed fruit consumption, habit strength, intention, attitudes, social influences, self-efficacy expectations and socio-demographics.

#### Demographics

Gender, age, and highest completed educational level were inquired after. Educational level was categorized into ‘low’ (elementary education, medium general secondary education, preparatory vocational school, or lower vocational school), ‘medium’ (higher general secondary education, preparatory academic education, or medium vocational school) and ‘high’ (higher vocational school or university level).

*Attitude* was assessed by six items pertaining to the outcomes concerning eating at least two pieces of fruit per day using four point scales: eating two fruits a day is tasty, pleasant, healthy, expensive, important, inconvenient (Cronbach’s α = .63). Answering options were very pleasant (4), pleasant (3), somewhat pleasant (2), not pleasant (1).

*Social influence beliefs* were assessed by three questions about norm, modeling and support. Social norm was assessed on a 7-point scale and assessed whether most people that were important to the participant believed that the participant should (definitely (7) - definitely not (1)) eat at least two pieces of fruit per day. Social modeling assessed perceived sufficient fruit consumption of people important to the participant, using a 5-point scale (almost everybody (5) - almost nobody (1)). Social support from important others to eat at least two pieces of fruit per day was assessed using a 5-point scale (never support me (5) - always support me (1)). Since these three items assessed different types of social influence [15], they were entered separately in the regression equations.

*Self-efficacy expectations* were measured by four items on a 7-point scale and asked to what extent respondents thought they would be (certainly not able (1) – certainly able (7)) to eat at least two pieces of fruit per day: during the week, during the weekend, when very busy, and during the winter months (α = 0.91).

*Intention* was measured by two items. The first item asked to what extent respondents intended to eat at least two pieces of fruit per day, and the second asked respondents to what extent they intended to eat two pieces of fruit per day in the next month. For both questions, answering options ranged from ‘I definitely do not intend to’ (1) to ‘I definitely intend to’ (7). Reliability of the intention measure was high (*r* = 0.93).

*Habit strength* was assessed at baseline and after 4 weeks (T2) using an abbreviated version of Verplanken & Orbell’s Self-Reported Habit Index (SRHI) [17], also used by Haustein et al. [35], which assesses respondents’ responses to the statements whether eating fruits is something that: I do often, that I do automatically, I do without thinking, belongs to my daily routine, it would cost me effort not to eat fruits, is something that is typically me (α = .95).

*Fruit consumption* was assessed at baseline (T1) and at follow-up after 4 weeks (T2) and after 8 weeks (T3), using a validated Dutch method [36] consisting of two items assessing: 1) the amount of days in a week the respondent usually eats fruit (0 to 7), and 2) the amount of fruit the respondent averagely consumes on each of these fruit eating days. Multiplying the responses to these two questions yields the amount of fruit consumed during a week (Spearman correlation coefficients with two 24-hour consumption recalls = 0.68 for men, 0.75 for women; correct tertile classification = 52%) [36]. Baseline assessment was used as an indicator of previous behavior.

### Statistical analyses

Analyses were conducted using SPSS 19. Frequencies, t-tests and logistic regression analyses were conducted to describe the characteristics of the sample. Correlation analysis was used to assess temporal stability of the constructs. Second, linear regression analyses were used to assess whether previous behavior and habit added unique variance in the explanation of fruit consumption after 4 and 8 weeks. In model 1 we entered all socio-demographic variables. In model 2 we added attitudes, social influences, self-efficacy, and intention. In model 3 we added habit strength. In model 4 we added baseline fruit consumption without the inclusion of habit strength, and in model 5 we entered both baseline fruit consumption and habit strength. Since habit and fruit consumption are highly correlated, variance inflation factors (VIF) and tolerance values were examined to assess multicollinearity. All VIF and tolerance values were well within the recommended range (all VIF < 4, tolerance > 0.20; [37]). Third, to assess mediation effects, we calculated the total effect of the independent variable on fruit consumption at follow- up after 4 and 8 weeks (c-coefficient). The effect of the mediator on this association was examined by using the products-of-coefficient method [38]. In this method, the effect of the independent variable on the theoretical mediator is calculated (a-coefficient), followed by the calculation of the association of the theoretical mediators on fruit consumption at follow-up after controlling for the independent variable at baseline (b-coefficient). The mediated effect (a*b) provides an estimate of the relative strength of the mediation effect. Bootstrapped 95% confidence intervals were calculated to assess the significance of the mediation effect (using 5000 bootstrap samples; [39]). All models were adjusted for baseline characteristics (i.e. gender, age, educational level) and psychosocial variables (i.e. attitude, self-efficacy, intention, social modeling, social support and social norm), and logistic regression analysis was used to assess which of these variables were associated with drop-out. All predictors were standardized to obtain z-scores, and Fischer’s z’ transformations were used to assess differences between correlations and differences between proportions of explained variance [40]. A power analysis (effect size (*f*^2^) = 0.05; power: .80; number of predictors (11), *p* < .05) revealed that at least 345 respondents would be needed for the analysis. Post hoc analysis (*N* = 434) reveal a power of .96.

## Results

### Characteristics of the sample

At baseline 574 respondents participated with an average age of 47 (*SD* = 15.98); 46.7% were female. Mean fruit consumption per week at baseline was 8.3 pieces per week. After four weeks (T2) this was 7.9 pieces of fruit per week, and after eight weeks this was 8.2 pieces of fruit per week. Mean fruit consumption differed significantly per habit level. Based on a baseline median split, we created two groups, one with a relatively low score (*M* = 3.07, *SD* = 1.02; *N* = 272) and one with a relatively high score of habit strength (*M* = 6.02, *SD* = 0.68; *N* = 302). At baseline, in the low habit strength level group, the mean fruit consumption was 3.96 per week versus 12.35 in the high habit strength group (*t*(566) = 19.53, *p* < .001). These results were 3.99 and 11.42 at T2 (*t*(489) = 17.22, *p* < .001) and 4.51 and 11.58 at T3 (*t*(426) = 14.51, *p* < .001). Logistic regression analyses revealed that the only baseline predictor of attrition at T2 (*N* = 76) and T3 (*N* = 64) was being younger (*β* = -0.47, *p* < .001; *β* = -0.22, *p* < .05).

### Stability of habit and previous behavior

In order to assess the stability of habit over time and previous behavior over time, we assessed the correlations at baseline (T1) and 4 weeks later (T2). Table 1 depicts the correlations of habit and behavior (Note, habit was not assessed at 8 weeks). The results revealed a correlation of the two habit assessments of .89. The correlation coefficients between baseline fruit consumption and consumption at four weeks and eight weeks follow-up were .80 and .76 respectively. Thus, over a period of 4 weeks, habit strength was more stable than actual fruit consumption (*Z* = 6.27, *p* < .001). In addition, baseline habit strength correlated with fruit consumption at baseline, and at four and eight weeks, with coefficients of .72, .69 and .64 respectively.

**Table 1:** Correlations of demographic factors, motivational factors, habit strength and fruit consumption

	**Gender**	**Age**	**Education**	**Attitude**	**Self-efficacy**	**Modeling**	**Support**	**Norm**	**Intention**	**HS T1**	**FC T1**	**HS T2**	**FC T2**
**Gender**													
**Age**	.167^***^												
**Education**	.212^***^	-.026											
**Attitude**	-.069	.150^***^	.109^**^										
**Self-efficacy**	-.018	.225^***^	.045	.713^***^									
**Modeling**	.038	.199^***^	.089^*^	.221^***^	.237^***^								
**Support**	.116^**^	.010	.034	-.015	.005	.290^***^							
**Norm**	.167^***^	.009	.018	-.176^***^	-.154^***^	.110^**^	.423^***^						
**Intention**	-.110^**^	-.011	.057	.491^***^	.582^***^	.134^**^	.157^***^	.127^**^					
**HS T1**	-.064	.180^***^	.089^**^	.698^***^	.774^***^	.213^***^	-.024	-.246^***^	.479^**^				
**FC T1**	-.056	.229^***^	.106^*^	.565^***^	.625^***^	.174^***^	-.081^‡^	-.201^***^	.420^**^	.715^***^			
**HS T2**	-.007	.165^***^	.134^**^	.701^***^	.737^***^.	.198^***^	-.009	-.188^***^	.488^**^	.894^***^	.699^***^		
**FC T2**	-.030	.210^***^	.091^*^	.559^***^	.604^***^	.142^**^	-.082^‡^	-.133^**^	.393^**^	.694^***^	.797^***^	.718^***^	
**FC T3**	-.080^‡^	.245^***^	.072	.517^***^	.572^***^	.129^**^	-.090^‡^	-.194^***^	.363^**^	.637^***^	.755^***^	.675^***^	.799^***^

FC = Fruit consumption; HS = Habit strength; ^‡^*p* < .10; ***p < .001; **p < .01; *p < .05.

### Contribution of previous behavior and habit strength besides motivational and demographic factors

We furthermore assessed whether baseline previous behavior and habit strength each explained unique variance after controlling for socio-demographic and motivational factors assessed at baseline (see Table 2). Concerning T2 fruit consumption, model 1 (with only baseline socio-demographic factors) explained 5.5% of the variance in predicting fruit consumption at T2, and revealed that an older age, higher education and female gender predicted fruit consumption. In model 2, motivational factors were added. The results showed that - besides older age - positive attitude, high self-efficacy, low social support (*p* = .07) and a high intention to eat more fruit (*p* = .08) were (marginally) significant predictors, explaining 40.7% of the variance of fruit consumption at T2. In model 3, habit strength was added to model 2. The results of model 3 showed that older age, positive attitude (*p* = .06), low social support and habit strength were significant predictors, explaining 50% of the variance. In model 4, baseline fruit consumption was added to model 2 (while habit strength was excluded) and was found to be a significant predictor besides attitude and self-efficacy, with 65.4% of the variance explained. This difference in added explained variance between habit strength (9.3%) and baseline fruit consumption (24.7%) was statistically significant (*Z* = 4.88, *p* < .001). Finally, both habit strength and baseline fruit consumption were included in model 5 increasing the explained variance up to only 66.4%, implying that habit hardly added any explained variance to the contribution of previous behavior (66.4% versus 65.4%; *Z* = 1.65, *p* = .10). Besides habit strength and baseline consumption, there were no other significant predictors in model 5.

**Table 2:** Contribution of previous behavior and habit strength besides cognitions and socio-demographics

**Fruit consumption T2**	**Model 1**		**Model 2**		**Model 3**		**Model 4**		**Model 5**	
	**β**	**p-value**	**β**	**p-value**	**β**	**p-value**	**β**	**p-value**	**β**	**p-value**
**Model 1**										
Gender	-.09	.03	-.02	.51	-.02	.62	-.01	.89	-.01	.92
Age	.23	<.01	.09	.01	.08	.02	.01	.83	.01	.74
Education	.11	.01	.06	.11	.04	.18	.03	.22	.03	.25
**Model 2**										
Attitude			.23	<.01	.09	.06	.11	<.01	.07	.09
Self-efficacy			.36	<.01	.09	.13	.12	<.01	.04	.43
Social modeling			-.01	.69	-.02	.49	-.03	.27	-.03	.24
Social support			-.07	.07	-.09	.02	-.03	.38	-.04	.23
Social norm			-.04	.30	.03	.36	.02	.50	.04	.17
Intention			.08	.08	.04	.30	-.01	.90	-.01	.80
**Model 3**										
Habit strength					.53	<.01	-	-	-	-
**Model 4**										
Previous behavior							.67	<.01	-	-
**Model 5**										
Habit strength									.20	<.01
Previous behavior									.60	<.01
**R**^**2**^	.06	<.01	.41	<.01	.50	<.01	.65	<.01	.66	<.01
**Fruit consumption T3**	**Model 1**		**Model 2**		**Model 3**		**Model 4**		**Model 5**	
	**β**	**p-value**	**β**	**p-value**	**β**	**p-value**	**β**	**p-value**	**β**	**p-value**
**Model 1**										
Gender	-.16	<.01	-.08	.04	-.08	.04	-.07	.03	-.07	.03
Age	.27	<.01	.16	<.01	.15	<.01	.08	.02	.08	.02
Education	.11	.02	.05	.17	.04	.22	.03	.40	.03	.40
**Model 2**										
Attitude			.17	<.01	.06	.24	.08	.08	.06	.19
Self-efficacy			.34	<.01	.12	.07	.11	.04	.07	.23
Social modeling			-.02	.71	-.03	.51	-.02	.46	-.03	.43
Social support			-.04	.33	-.05	.19	-.01	.87	-.01	.77
Social norm			-.13	<.01	-.06	.14	-.05	.19	-.04	.31
Intention			.10	.05	.06	.19	-.01	.76	.01	.82
**Model 3**										
Habit strength					.42	<.01	-	-	-	-
**Model 4**										
Previous behavior							.61	<.01	-	-
**Model 5**										
Habit strength									.09	.11
Previous behavior									.58	<.01
**R**^**2**^	.08	<.01	.38	<.01	.44	<.01	.59	<.01	.59	<.01

Table 2 also shows the result of similar analyses for predicting fruit consumption after 8 weeks (T3). Model 1 explained 8% of the variance in predicting fruit consumption after 8 weeks (T3) with older age, female gender and high education level as significant predictors. Model 2 explained 38% of the variance with older age, female gender, a positive attitude, high self-efficacy, negative social norms and a high intention as significant predictors. Model 3 explained 44.2% of the variance, with habit strength, high self-efficacy (*p* = .07), higher age and female gender as (marginal) significant predictors. In model 4 (including baseline behavior but not habit strength) baseline fruit consumption, positive attitudes (*p* = .08), high self-efficacy, higher age and female gender were (marginal) significant, explaining 59.2% of the variance. In line with the results for T2, the inclusion of baseline consumption led to significantly more added explained variance for predicting T3 fruit consumption (21.2%) than the inclusion of habit strength (6.2%; *Z* = 5.16, *p* < .001). Finally, baseline fruit consumption was entered in model 5, along with habit strength. The former was the only significant predictor besides older age and female gender; habit did not contribute significantly. Model 5’s overall contribution was not significantly higher than that of model 4 (59.4% versus 59.2%; *Z* < 1, *p* = .92).

Furthermore, we reran the analyses with a slightly adapted version of the SRHI by removing two items that assess behavioral frequency (i.e. “I do often” and “belongs to my daily routine”). The results were similar to the ones described above with slightly smaller effect sizes of habit strength. The association between habit strength and fruit consumption at T2 was .42 without including previous behavior (model 3 in Table 2; R^2^ = .48), and .16 after including previous behavior (model 5 in Table 2; R^2^ = .65). The association between habit strength and fruit consumption at T3 was .34 without including previous behavior (model 3 in Table 2; R^2^ = .43) and .08 after including previous behavior (model 5 in Table 2; R^2^ = .59).

### Mediation effects

**Figure 1:**
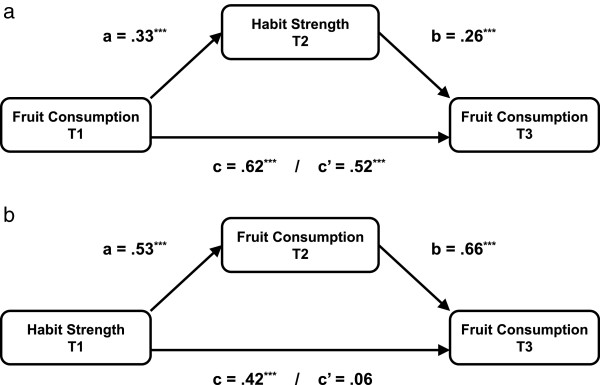
**Mediation effects. (a)** Mediation of the previous behavior – future behavior relationship **(b)** Mediation of the habit – behavior relationship.

We furthermore assessed potential mediation by habit strength in the association between previous and future behavior. Figure 1a depicts the total effect (c-coefficient), direct effect (c’-coefficient, when adjusted for habit strength) of baseline fruit consumption on future fruit consumption (T3) and the mediation effect (ab) of habit strength (T2). The results showed that the effect of baseline fruit consumption (c = .62) on future fruit consumption at T3 was partly mediated by habit strength (c’ = .52). The mediation effect of habit strength was significant (ab = .09; bootstrapped 95% CI =0.05 – 0.14) and accounted for about 14% of the total effect.Since the regression analyses that were done to assess the contribution of habit strength and previous behavior implied mediation of habit via previous behavior, we also tested this reversed mediation pattern. Figure 1b shows the mediation effect of previous behavior (T2) on the association between habit strength (T1) and fruit consumption (T3). The results reveal a significant total effect (c = .42) of baseline habit strength on fruit consumption after eight weeks (T3). The direct effect, after controlling for previous behavior, was .06 and not significant. This mediation effect of previous behavior on the association between habit strength (T1) and fruit consumption (T3) was significant (ab = .35; bootstrapped 95% CI = 0.28 – 0.45) and accounted for 83% of the total effect.

In sum, we found some albeit weak support for the hypothesis that the effect of previous behavior on future fruit consumption would be mediated by habit strength. We did find, however, that previous behavior strongly mediated the habit strength – future behavior association.

## Discussion

The influence of previous behavior and habits on current behavior is a topic that has attracted considerable attention in general, as well as for understanding nutrition behavior in particular [8,13,41]. The importance of adding both habit and previous behavior as important constructs to socio-cognitive models has been outlined before, yet not always with supporting evidence from longitudinal studies that included both constructs. Our study builds on earlier research and our findings reveal the following conclusions.

First, as predicted we found support for our hypothesis that habit assessments were slightly more stable over time than assessments of behavior. Yet, only 80% of habit strength could be predicted by habit strength one month earlier. Only 64% of fruit consumption could be predicted by fruit consumption one month earlier, which was further reduced to 57% if baseline fruit consumption was used to predict fruit consumption two months later. These findings suggest less temporal stability over such a small period of time (two months) than we had expected.

Second, contrary to assumptions of TPB, habit and previous behavior had a unique contribution in explaining future fruit consumption at T2, supporting earlier findings (e.g. [15,28,42]), and the assumptions of integrative models that acknowledge a direct path of habit and previous behavior [32,43]. The variance explained by motivational factors corresponds with reports from previous studies suggesting that motivational variables explain approximately 30 to 40% of the explained variance in self-reported behavior [44-46]. In our study, the model with motivational constructs explained 41% and 38% of the behavioral variance at T2 and T3. The addition of previous behavior and habit increased the proportion of explained variance to 66% and 59% at T2 and T3. Our findings corroborate with those reported by several other studies for similar and other health behaviors (see e.g. [10,11,15,16,43,47-49]), although some other studies failed to find such directs paths of previous behavior and/or habit (see e.g. [20]). Our results also revealed that the influences of attitude and self-efficacy were still significant when intentions were included in the model, a finding that is in contrast with predictions made by the Theory of Planned Behavior for attitudes. As a matter of fact, the unique contribution of intentions was much lower than those of attitudes and self-efficacy. This clearly implies that more research is needed to determine when attitudes and self-efficacy may have a more prominent role in predicting behavior than intentions.

Third, motivational factors failed to have a significant contribution in explaining future behavior when previous behavior and habits were entered in the model. When only previous behavior was entered in the model, however, contributions of attitude and self-efficacy were significant. This may support the idea that habits influence the development of attitude and self-efficacy beliefs. We posit a reciprocal relationship between habits and cognitions: cognitions may influence the development of behavior and/or behavior and habits influence cognitions. Additionally, habits may also reinforce beliefs by preventing cognitive dissonance [50]. The latter issue was not the focus of this study, but clearly is noteworthy to explore further in order to obtain a better understanding of these potential consequences of habit formation.

Fourth, our results showed that the effect of habit strength on future behavior was to a large extent mediated by previous behavior. The mediated effect of previous behavior accounted for 58% and 83% of the total effect of habit strength on future behavior at T2 and T3 respectively. Research using larger time intervals is needed to assess whether similar findings will be observed under these circumstances. Our results (for T3) were not similar to those reported by Verplanken [28], who found that habit and past snacking frequency independently predicted later snacking behavior after one week (controlling for TPB variables). In addition, Verplanken found that habit fully mediated the effect of previous behavior on later behavior. Their study, however, used different measurements for assessing past and future behavior and had a time interval of only one week. A study by Knussen and colleagues [48] found support for significant contribution of both previous behavior and habit, whereas a study by Bamberg et al. [51] failed to find any support for the hypothesis that the effect of previous behavior on future behavior is due to habit. When excluding the items in the habit scale that referred to behavior frequency, we found similar patterns which supports findings outlined by Verplanken & Orbel [17]; Gardner, de Bruin & Lally [8] and Gardner, Abraham, Lally & De Bruin [52].

Several studies have analyzed the predictive value of habits. Yet, in order to be able to accept habit as a valid explanation of residual variance, two conditions need to be met [23]. First, habit should be measured with an instrument independent of past behavioral frequency. By the use of the SRHI and by including separate measurements of previous behavior, this condition was met in our study. Second, habit should mediate the relationship between prior and later behavior. Although not all effect on later behavior was exerted through habit (14%), our results showed that habit offered a partial solution to the residual variance problem, with small to medium effect sizes, which are common [38]. Hence, the results of our study yield support to the conclusion that previous behavior and habits as constructs that can influence behavior beyond cognitions.

Despite our longitudinal design, some study limitations should be noted. First, our comparison of temporal stability between habit strength and previous behavior might be biased, since our measure of habit included several questions and our measure of previous behavior only one. Second, drawing final conclusions regarding the causality of the mediation effects is complex since habit and previous behavior are likely to be entangled in an ongoing reinforcing circle. Habits are formed by previous behavior and once habits are formed they trigger subsequent execution of the behavior. Hence, the results of the mediation analyses should be interpreted with caution. Third, the way habit influences behavior might differ for other behaviors and replication is needed. Fourth, recent research demonstrated the importance of habit strength and implicit associations [10] indicating substantial relevance of positive implicit associations to strengthen the habit strength—health behavior relationship. This finding suggests a replication of the current study using both explicit and implicit assessments of beliefs. Finally, our results could have been slightly different when other constructs or measurements of core social cognitive constructs were used. For instance, once could argue that results could have been different when perceived behavioral control was used instead of self-efficacy. Yet, according to Fishbein & Ajzen [22] and Ajzen [53], perceived behavioral control (PBC) is conceptually similar to self-efficacy. Other authors, however, have argued that they are conceptually distinct [54]. Since previous studies have shown that self-efficacy explains most of the variance in intentions in comparison to PBC [44,55-57], we used the self-efficacy construct as proposed [58]. Lastly, the results of the mediation analysis presented in Figure 1b may be partly explained by the shared method variance as fruit consumption at T2 and T3 are more similar to each other than habit at T1 and fruit consumption at T3, increasing the likelihood of indirect effects.

What are potential research implications of our findings? In terms of model development our results support findings suggesting the inclusion of measurements of previous behavior and habits. Our results did not suggest a unique contribution of habit strength above the inclusion of previous behavior at T3, thus supporting an earlier conclusion by Triandis [59] that measures of past behavioral frequency may be an adequate proxy for habits; yet replication of these findings for other behaviors and different time intervals are needed before final conclusions can be drawn. One explanation may be that when both constructs are used, the SRHI may not be capturing automaticity sufficiently [51]. Yet, our findings suggested the same findings when only the automaticity items of the SRHI were included. Future research needs to assess the conditions that determine direct influences of both previous behavior and habit, since these influences may differ per type of health behavior, per context stability in which the behavior is performed, and per time frame used for predicting future behavior. Our findings do not imply that habit is less important than previous behavior. If a person consistently eats fruit on the same occasion, one could conclude that habits may have no additional variance, whereas the behavior may have been very well prompted automatically by habits. On the other hand, if a person eats fruits deliberately on the same occasion, the engagement of the person in this behavior may also reflect the results of preference and a deliberate choice for this behavior. Our hypothesis is that previous behavior can play a unique and meaningful role in explaining behavior when the behavior is not fully under control of habits that automatically prompt the behavior. Additionally, we hypothesize that the level of engagement in previous behavior may reflect a conscious decision based on a person’s preference for a particular behavior, whereas a habit reflects more an automatic reaction that may have become linked to certain stimuli. Clearly, our findings outline a need for a further analysis of the habit formation processes and the roles of previous behavior and habits.

What are potential implications of our findings for behavior change interventions? Our results support earlier findings suggesting the importance of previous behavior and habit in understanding eating behaviors [8,13]. The engagement in a particular behavior may reflect a person’s preference for this behavior as well that the behavior may have become an automatism. Our results also suggest that fruit consumption habits may not be as stable as previously thought thus suggesting more potential to change them. Educational interventions need to address the importance of these factors more explicitly besides addressing only motivational components [13,60]. This implies the importance of regular monitoring of fruit consumption behavior to increase awareness of one’s behavior and to be able to signal reduction in fruit consumption timely before not eating fruits has become a habit. Several strategies can be chosen which are not mutually exclusive, which need to focus on unfreezing old habits and the promotion of habit formation of a new behavior, supporting context-dependent repetition of this behavior, and the facilitation of automaticity for these new behaviors; this implies restructuring the personal environment and enabling alternative responses to situational cues [61] as well as motivation for self-control strategies to block triggering old habits [62]. Habitual behavior is likely to occur as a response to cues that prompt the habit [4,13]. Unfreezing habits requires unlinking cues that prompt and reinforce habitual behavior, thus increasing the likelihood of alternative responses [62]. One strategy is to target social-environmental factors and cues that prompt habitual behavior [6,7,11-13,20,63-66]. For fruit consumption this type of strategy may not be the only type of intervention that will be sufficient. If people need to change their environment, for instance by having more fruits easily available in the house, this still requires motivation to change their environment which will be likely dependent on their motivation to change their dietary habits. Another strategy is to increase intrinsic motivation towards realizing health, as behavior change is more likely when people are motivated by intrinsic rather than extrinsic motivations [67] as well as to increase the salience of the consequences of (habitual) behaviors that may impede this goal. The use of such critical situations has shown to be effective in changing automatic reactions. Macrae and Johnston [68], for instance, demonstrated that when an automatic reaction is inconsistent with another goal, this may reduce the likelihood of automatic goal pursuit. In addition, previous social cognitive approaches for changing habitual behaviors may have failed because they did not include sufficient time to change existing belief structures [6,69]. Acting in non-habitual ways requires a personal decision to act differently and to override habitual responses in memory [70]. Using implementation intentions can change unwanted habits [71] but still require motivational efforts [62]. In addition, cognitions that are associated with habitual behaviors are strongly anchored within a persons’ belief system. People with strong habits may not easily be motivated to change, because of potentially biased information processing [13], but also because habits may reinforce beliefs about a particular behavior. Consequently, motivational approaches imply raising awareness about detrimental consequences of habits, awareness of cues that may trigger them, and – as outlined by Rothman and colleagues [62] - high levels of self-control by using for instance (vigilant) monitoring of the behavior, ‘risky’ cues and self-instructions on how to respond to these cues. Rothman, Sheeran & Wood [62] also suggest other types of strategies, for instance aimed at addressing implicit attitudes that may prompt behavior automatically, such as evaluative conditioning, association training and approach/avoidance training.

## Conclusions

Several conclusions can be drawn. First, while our data supports the importance of both habit and previous behavior, the data also revealed that in our study previous behavior was a more direct predictor than habit: the effect of habit strength on future behavior was to a large extent mediated by previous behavior. Our results also showed that habit and behavior were only moderately stable over a four week period. Second, health behavior models need to include previous behavior and habits as important predictors. Whether both factors need to be assessed simultaneously is subject for further research. Third, motivational factors failed to have a significant contribution in explaining future behavior when previous behavior and habits were entered in the model. We posit a reciprocal relationship between habits and cognitions. Third, further research is needed to understand the conditions that may lead to significant contributions of attitude and self-efficacy beyond intentions. Fourth, the effect of habit strength on future behavior was to a large extent mediated by previous behavior. Finally, in order to change (nutrition) habits, interventions require strategies that impact on several factors, such as (social environmental) cues, awareness, motivation and self-monitoring [13,72] that take into account a reciprocal relationship between habits and cognitions.

## Competing interests

Hein de Vries is the scientific director of Vision2Health, a company that licenses evidence-based innovative computer-tailored health communication tools. No other authors reported conflicts of interest.

## Authors’ contribution

HdV participated in the design of the study and writing of the paper. LvO and LL were involved in the questionnaire development, data cleaning and writing of the paper. SE and MvS were both involved in the data analysis and writing of the paper. All authors read and approved the final manuscript.

## Pre-publication history

The pre-publication history for this paper can be accessed here:

http://www.biomedcentral.com/1471-2458/14/730/prepub

## References

[B1] Paolini M Sapone A Canistro D Antonelli MA Chieco P Diet and risk of cancer Lancet 2003 361 9353 257 258 10.1016/S0140-6736(03)12286-6 12547566

[B2] Key TJ Allen NE Spencer EA Travis RC The effect of diet on risk of cancer Lancet 2002 360 9336 861 868 10.1016/S0140-6736(02)09958-0 12243933

[B3] van Rossum CTM Fransen HP Verkaik-Kloosterman J Buurma-Rethans EJM Ocké MC Dutch National Food Consumption Survey 2007–2010: Diet of children and adults aged 7 to 69 years 2011 RIVM report 350050006: National Institute for Public Health and the Environment: Ministry of Health, Welfare and Sport

[B4] Orbell S Verplanken B The automatic component of habit in health behavior: habit as cue-contingent automaticity Health Psychol 2010 29 4 374 383 20658824 10.1037/a0019596

[B5] Ouellette JA Wood W Habit and intention in everyday life: The multiple processes by which past behavior predicts future behavior Psychol Bull 1998 124 1 54 74

[B6] Webb TL Sheeran P Does changing behavioral intentions engender behavior change? A meta-analysis of the experimental evidence Psychol Bull 2006 132 2 249 268 16536643 10.1037/0033-2909.132.2.249

[B7] Aarts H Verplanken B van Knippenberg A Predicting behavior from actions in the past: Repeated decision making or a matter of habit? J Appl Soc Psychol 1998 28 15 1355 1374 10.1111/j.1559-1816.1998.tb01681.x

[B8] Gardner B de Bruijn GJ Lally P A systematic review and meta-analysis of applications of the Self-Report Habit Index to nutrition and physical activity behaviours Ann Behav Med 2011 42 2 174 187 10.1007/s12160-011-9282-0 21626256

[B9] Verplanken B Aarts H van Knippenberg A Moonen A Habit versus planned behaviour: a field experiment Br J Soc Psychol 1998 37 Pt 1 111 128 9554090 10.1111/j.2044-8309.1998.tb01160.x

[B10] de Bruijn GJ Understanding college students’ fruit consumption. Integrating habit strength in the theory of planned behaviour Appetite 2010 54 1 16 22 10.1016/j.appet.2009.08.007 19712718

[B11] de Bruijn GJ Kremers SPJ De Vet E De Nooijer J Van Mechelen W Brug J Does habit strength moderate the intention-behaviour relationship in the theory of planned behaviour? The case of fruit consumption Psychol Health 2007 22 8 899 916 10.1080/14768320601176113

[B12] Reinaerts E de Nooijer J Candel M de Vries N Explaining school children’s fruit and vegetable consumption: the contributions of availability, accessibility, exposure, parental consumption and habit in addition to psychosocial factors Appetite 2007 48 2 248 258 10.1016/j.appet.2006.09.007 17109996

[B13] Riet JV Sijtsema SJ Dagevos H De Bruijn GJ The importance of habits in eating behaviour. An overview and recommendations for future research Appetite 2011 57 3 585 596 10.1016/j.appet.2011.07.010 21816186

[B14] Brug J van Lenthe FJ Kremers SP Revisiting Kurt Lewin: how to gain insight into environmental correlates of obesogenic behaviors Am J Prev Med 2006 31 6 525 529 10.1016/j.amepre.2006.08.016 17169715

[B15] de Vries H Backbier E Kok G Dijkstra M The impact of social influences in the context of attitude, self-efficacy, intention, and previous behavior as predictors of smoking onset J Appl Soc Psychol 1995 25 3 237 257 10.1111/j.1559-1816.1995.tb01593.x

[B16] Lechner L de Vries H Offermans N Participation in a breast cancer screening program: influence of past behavior and determinants on future screening participation Prev Med 1997 26 4 473 482 10.1006/pmed.1997.0161 9245669

[B17] Verplanken B Orbell S Reflections on past behavior: a self-report index of habit strength J Appl Soc Psychol 2003 33 6 1313 1330 10.1111/j.1559-1816.2003.tb01951.x

[B18] Hagger MS Chatzisarantis N Biddle SJ The influence of self-efficacy and past behaviour on the physical activity intentions of young people J Sports Sci 2001 19 9 711 725 10.1080/02640410152475847 11522147

[B19] Jackson C Smith RA Conner M Applying an extended version of the theory of planned behaviour to physical activity J Sports Sci 2003 21 2 119 133 10.1080/0264041031000070976 12630791

[B20] Verplanken B Melkevik O Predicting habit: The case of physical exercise Psychol Sport Exerc 2008 9 1 15 10.1016/j.psychsport.2007.01.002

[B21] Ajzen I The theory of planned behavior Organ Behav Hum Decis Process 1991 50 179 211 10.1016/0749-5978(91)90020-T

[B22] Fishbein M Ajzen I Prediction and Change of Behavior: The Reasoned Action Approach 2010 New York: Psychology Press

[B23] Ajzen I Residual effects of past on later behavior: Habituation and reasoned action perspectives Pers Soc Psychol Rev 2002 6 2 107 122 10.1207/S15327957PSPR0602_02

[B24] Bandura A Social Foundations of Thought and Action 1986 NJ: Englewood Cliffs

[B25] Conner M Abraham C Conscientiousness and the theory of planned behavior: toward a more complete model of the antecedents of intentions and behavior Pers Soc Psychol Bull 2001 27 11 1547 10.1177/01461672012711014

[B26] de Bruijn GJ Kremers SP Lensvelt-Mulders G de Vries H van Mechelen W Brug J Modeling individual and physical environmental factors with adolescent physical activity Am J Prev Med 2006 30 6 507 512 10.1016/j.amepre.2006.03.001 16704945

[B27] Norman P Smith L The theory of planned behaviour and exercise: An investigation into the role of prior behaviour, behavioural intentions and attitude variability Eur J Soc Psychol 1995 12 4 403 415

[B28] Rhodes RE Courneya KS Jones LW Translating exercise intentions into behavior: personality and social cognitive correlates J Health Psychol 2003 8 4 447 458 10.1177/13591053030084004 19127711

[B29] Rhodes RE Courneya KS Modelling the theory of planned behaviour and past behaviour Psychol Health Med 2003 8 1 57 69 10.1080/1354850021000059269 21888489

[B30] Verplanken B Beyond frequency: habit as mental construct Br J Soc Psychol 2006 45 Pt 3 639 656 16984725 10.1348/014466605X49122

[B31] van Osch L Beenackers M Reubsaet A Lechner L Candel M de Vries H Action planning as predictor of health protective and health risk behavior: an investigation of fruit and snack consumption Int J Behav Nutr Phys Act 2009 6 69 10.1186/1479-5868-6-69 19825172 PMC2770554

[B32] Centrale Commissie Mensgebonden Onderzoekhttp://www.ccmo.nl

[B33] Verenigde Commissies Mensgebonden Onderzoekhttps://www.vcmo.nl/wmo/niet-wmo-plichtig-onderzoek/

[B34] de Vries H Kremers SP Smeets T Brug J Eijmael K The effectiveness of tailored feedback and action plans in an intervention addressing multiple health behaviors Am J Health Promot 2008 22 6 417 425 10.4278/ajhp.22.6.417 18677882

[B35] Haustein S Klöckner CA Blöbaum A Car use of young adults: The role of travel socialization Transp Res 2009 12 168 178

[B36] van den Brink CL Ocke MC Houben AW van Nierop P Droomers M Validering van standaardvraagstelling voeding voor Lokale en Nationale Monitor Volksgezondheid 2005 Bilthoven: RIVM

[B37] O’brien RM A caution regarding rules of thumb for variance inflation factors Qual Quant 2007 41 5 673 690 10.1007/s11135-006-9018-6

[B38] MacKinnon DP Introduction to Statistical Mediation Analysis 2008 New York: Erlbaum and Taylor Francis Group

[B39] Preacher KJ Hayes AF SPSS and SAS procedures for estimating indirect effects in simple mediation models Behav Res Methods Instrum Comput 2004 36 4 717 731 10.3758/BF03206553 15641418

[B40] Meng XL Rosenthal R Rubin DB Comparing correlated correlation coefficients Psychol Bull 1992 111 1 172 175

[B41] Eagly AH Chaiken S The Psychology of Attitudes 1993 1 Orlando, FL: Harcourt Brace Jovanovich College Publishers

[B42] Conner M Armitage CJ Extending the theory of planned behavior: A review and avenues for further research J Appl Soc Psychol 1998 28 15 1429 1464 10.1111/j.1559-1816.1998.tb01685.x

[B43] Norman P Conner M Bell R The theory of planned behaviour and exercise: Evidence for the moderating role of past behaviour Br J Health Psychol 2000 5 249 261 10.1348/135910700168892 16480555

[B44] Armitage CJ Conner M Efficacy of the Theory of Planned Behaviour: a meta-analytic review Br J Soc Psychol 2001 40 Pt 4 471 499 11795063 10.1348/014466601164939

[B45] Godin G Kok G The theory of planned behavior: A review of its applications to health-related behaviors Am J Health Promot 1996 11 2 87 98 10.4278/0890-1171-11.2.87 10163601

[B46] Perugini M Bagozzi RP The distinction between desires and intentions Eur J Soc Psychol 2004 34 1 69 84 10.1002/ejsp.186

[B47] Godin G Amireault S Belanger-Gravel A Vohl M-C Perusse L Guillaumie L Prediction of daily fruit and vegetable consumption among overweight and obese individuals Appetite 2010 54 3 480 484 10.1016/j.appet.2010.01.018 20138945

[B48] Knussen C Yule F MacKenzie J Wells M An analysis of intentions to recycle household waste: The roles of past behaviour, perceived habit, and perceived lack of facilities J Environ Psychol 2004 24 2 237 246 10.1016/j.jenvp.2003.12.001

[B49] Kremers SP van der Horst K Brug J Adolescent screen-viewing behaviour is associated with consumption of sugar-sweetened beverages: the role of habit strength and perceived parental norms Appetite 2007 48 3 345 350 10.1016/j.appet.2006.10.002 17126451

[B50] Festinger L A Theory of Cognitive Dissonance, Volume 2 1962 Redwood City, CA: Stanford University Press

[B51] Bamberg S Ajzen I Schmidt P Choice of travel mode in the theory of planned behavior: the roles of past behavior, habit, and reasoned action Basic Appl Soc Psych 2003 25 175 187 10.1207/S15324834BASP2503_01

[B52] Gardner B Abraham C Lally P de Bruijn G-J Towards parsimony in habit measurement: Testing the convergent and predictive validity of an automaticity subscale of the Self-Report Habit Index Int J Behav Nutr Phys Act 2012 9 1 102 10.1186/1479-5868-9-102 22935297 PMC3552971

[B53] Ajzen I Martin Fishbein’s Legacy the Reasoned Action Approach Ann Am Acad Polit Soc Sci 2012 640 1 11 27 10.1177/0002716211423363 PMC3520136 23243315

[B54] Trafimow D Sheeran P Conner M Finlay KA Evidence that perceived behavioural control is a multidimensional construct: Perceived control and perceived difficulty Br J Soc Psychol 2002 41 1 101 121 10.1348/014466602165081 11970777

[B55] Manstead AS Eekelen SA Distinguishing between perceived behavioral control and self‒efficacy in the domain of academic achievement intentions and behaviors J Appl Soc Psychol 1998 28 15 1375 1392 10.1111/j.1559-1816.1998.tb01682.x

[B56] Terry DJ O’Leary JE The theory of planned behaviour: The effects of perceived behavioural control and self‒efficacy Br J Soc Psychol 1995 34 2 199 220 10.1111/j.2044-8309.1995.tb01058.x 7620846

[B57] White KM Terry DJ Hogg MA Safer sex behavior: The role of attitudes, norms, and control factors J Appl Soc Psychol 1994 24 24 2164 2192 10.1111/j.1559-1816.1994.tb02378.x

[B58] de Vries H Dijkstra M Kuhlman P Self-efficacy: the third factor besides attitude and subjective norm as a predictor of behavioural intentions Health Educ Res 1988 3 3 273 282 10.1093/her/3.3.273

[B59] Triandis HC Interpersonal Behavior 1977 CA: Brooks/Cole Publishing Company Monterey

[B60] Verplanken B Wood W Interventions to break and create consumer habits J Public Policy Mark 2006 25 1 90 103 10.1509/jppm.25.1.90

[B61] Lally P Gardner B Promoting habit formation Health Psychol Review 2013 7 (sup1 S137 S158

[B62] Rothman AJ Sheeran P Wood W Reflective and automatic processes in the initiation and maintenance of dietary change Ann Behav Med 2009 38 1 4 17 10.1007/s12160-009-9130-7 19787308

[B63] Sutton S Rutter DR, Quine L The Past Predicts the Future: Interpreting Behaviour-Behaviour Relationships in Social Psychological Models of Health Behaviour Social Psychology and Health: European Perspectives Edn 1994 Aldershot: Avebury 71 88

[B64] de Bruijn GJ van den Putte B Adolescent soft drink consumption, television viewing and habit strength. Investigating clustering effects in the Theory of Planned Behaviour Appetite 2009 53 1 66 75 10.1016/j.appet.2009.05.008 19463873

[B65] Brug J de Vet E de Nooijer J Verplanken B Predicting fruit consumption: cognitions, intention, and habits J Nutr Educ Behav 2006 38 2 73 81 10.1016/j.jneb.2005.11.027 16595285

[B66] Verplanken B Faes S Good intentions, bad habits, and effects of forming implementation intentions on healthy eating Eur J Soc Psychol 1999 29 5–6 591 604

[B67] de Ridder D de Wit J Adriaanse MA Making plans for healthy diet: The role of motivation and action orientation Eur J Soc Psychol 2009 39 4 622 630 10.1002/ejsp.560

[B68] Macrae CN Johnston L Help, I need somebody: Automatic action and inaction Soc Cogn 1998 16 4 400 417 10.1521/soco.1998.16.4.400

[B69] Hardeman W Johnston M Johnston DW Bonetti D Wareham NJ Kinmonth AL Application of the Theory of Planned Behaviour in behaviour change interventions: A systematic review Psychol Health 2002 17 2 123 158 10.1080/08870440290013644a

[B70] Wood W Neal DT The habitual consumer J Consum Psychol 2009 19 579 592 10.1016/j.jcps.2009.08.003

[B71] Adriaanse MA Gollwitzer PM De Ridder DTD de Wit JBF Kroese FM Breaking habits with implementation intentions: a test of underlying processes Pers Soc Psychol Bull 2011 37 4 502 513 10.1177/0146167211399102 21317315

[B72] Wood W Witt MG Tam L Changing circumstances, disrupting habits J Pers Soc Psychol 2005 88 6 918 933 15982113 10.1037/0022-3514.88.6.918

